# Mucosal Delivery of Fusion Proteins with *Bacillus subtilis* Spores Enhances Protection against Tuberculosis by Bacillus Calmette-Guérin

**DOI:** 10.3389/fimmu.2018.00346

**Published:** 2018-03-12

**Authors:** Alastair Copland, Gil R. Diogo, Peter Hart, Shane Harris, Andy C. Tran, Mathew J. Paul, Mahavir Singh, Simon M. Cutting, Rajko Reljic

**Affiliations:** ^1^St George’s Medical School, London, United Kingdom; ^2^Lionex GmbH, Braunschweig, Germany; ^3^School of Biological Sciences, Royal Holloway University of London, Egham, United Kingdom

**Keywords:** tuberculosis, spores, vaccine, immunity, adjuvants

## Abstract

Tuberculosis (TB) is the most deadly infectious disease in existence, and the only available vaccine, *Bacillus Calmette-Guérin* (BCG), is almost a century old and poorly protective. The immunological complexity of TB, coupled with rising resistance to antimicrobial therapies, necessitates a pipeline of diverse novel vaccines. Here, we show that *Bacillus subtilis* spores can be coated with a fusion protein 1 (“FP1”) consisting of *Mycobacterium tuberculosis* (Mtb) antigens Ag85B, ACR, and HBHA. The resultant vaccine, Spore-FP1, was tested in a murine low-dose Mtb aerosol challenge model. Mice were primed with subcutaneous BCG, followed by mucosal booster immunizations with Spore-FP1. We show that Spore-FP1 enhanced pulmonary control of Mtb, as evidenced by reduced bacterial burdens in the lungs. This was associated with elevated antigen-specific IgG and IgA titers in the serum and lung mucosal surface, respectively. Spore-FP1 immunization generated superior antigen-specific memory T-cell proliferation in both CD4^+^ and CD8^+^ compartments, alongside bolstered Th1-, Th17-, and Treg-type cytokine production, compared to BCG immunization alone. CD69^+^CD103^+^ tissue resident memory T-cells (Trm) were found within the lung parenchyma after mucosal immunization with Spore-FP1, confirming the advantages of mucosal delivery. Our data show that Spore-FP1 is a promising new TB vaccine that can successfully augment protection and immunogenicity in BCG-primed animals.

## Introduction

In 2015, tuberculosis (TB) overtook HIV/AIDS as the leading cause of death due to infection (1). This statistic contrasts starkly with the fact that the only available TB vaccine, *Mycobacterium bovis* Bacillus Calmette-Guérin (BCG), is the most widely administered vaccine in history (2), having been developed almost a century ago. The proposed reasons for the failure of BCG to adequately protect against TB are many and varied. They include (i) BCG sub-strain heterogeneity (3), (ii) pre-exposure of the host to environmental non-tubercle mycobacteria (4), (iii) a failure to prevent pulmonary infection (5), and (iv) limited protection in adults compared to children (6). Despite these limitations, BCG is unlikely to be discontinued in clinical use. While its efficacy in many demographics is modest, there are accumulating data indicating that BCG may protect against non-TB diseases by training the innate immune system to respond non-specifically to diverse microbial threats (7, 8). A novel TB vaccine is therefore likely to supplement, rather than replace, BCG.

In 2011, the novel viral vector TB vaccine MVA85A, comprising Ag85A, was tested for safety and efficacy in a phase 2 clinical trial in South Africa, and it was found that parenteral administration of MVA85A in BCG-immunized infants offered no significant protection above that of BCG alone (9). The reasons for its failure are still unclear, since MVA85A protected against *Mycobacterium tuberculosis* (Mtb) in multiple animal models (10). But it is becoming increasingly apparent that the development pipeline for new TB vaccines will require technological diversity in order to maximize chances of success. In recent years, vaccines that are based upon particulate nano- or microscale delivery systems have made remarkable strides in both oncology and infectious diseases (11–13).

*Bacillus subtilis* is an environmental Gram-positive bacterium that is also found as a gut commensal in humans (14). Its spores have the desirable properties of being both safe and adjuvantic (15). But more importantly, they possess hydrophobic and electrostatic properties that allow them to readily bind protein antigens, making these spores pertinent to vaccine development as potential antigen delivery systems (16). The combination of intrinsic adjuvanticity and antigen-binding biophysical properties allows *B. subtilis* spores to act simultaneously as adjuvants and antigen carriers. Studies have shown that immunization of mice with *B. subtilis* spores coated with the influenza antigen M2e can induce strong antibody responses and protect against lethal challenge (17, 18). Similar findings have been observed in other immunization models, including immunogenicity against HIV and streptococci (19, 20). *B. subtilis* spores are thus an attractive platform for subunit vaccine enhancement.

We have previously shown that *B. subtilis* spores coated with TB antigens (21) or genetically engineered to express a TB antigen (22) can enhance protection against TB by BCG (prime-boost) in a mouse intranasal infection model. Although this provided a proof-of-principle framework for vaccine efficacy, the use of genetically modified components in a vaccine presents numerous regulatory barriers for clinical application (23). Here, we developed a novel TB vaccine—“Spore-FP1”—composed of *B. subtilis* spores non-covalently coated with a fusion protein (FP1) consisting of the antigens Ag85B, ACR and the epithelium-binding domain of HBHA (“FP1”). Ag85B and ACR were chosen to represent early and late stages of *Mtb* infection, respectively, while HBHA (heparin-binding domain only) was used for epithelial targeting in the lungs. Mucosal booster immunization with Spore-FP1 in BCG-primed mice enhanced protection in a low-dose aerosol Mtb challenge model, compared to BCG alone. The enhanced protection was concomitant with a wide array of boosted immunological parameters, including enhanced antigen-dependent T-cell proliferation and antibody production. Spore-FP1 is therefore a novel TB vaccine that has the potential to supplement pre-existing immunity conferred by BCG in human populations.

## Materials and Methods

### Ethics Statement

All animals were used with approval from St. George’s University of London Ethics Committee under an approved Home Office animal project license (70/7490) and used in accordance with the Animals (Scientific Procedures) Act 1986.

### Mice and Immunizations

Female C57BL/6 mice were obtained from Charles River, UK, and were between 8 and 12 weeks of age before experimental use. For all bacterial challenge or immunogenicity experiments (except lung T-cell analysis), mice were immunized with 5 × 10^5^ CFU BCG Pasteur (100 µL) subcutaneously or vehicle control. Intranasal booster immunizations consisted of 1 × 10^9^ *B. subtilis* spores coated with 10 µg FP1 in 40 µL volumes per animal per dose, or vehicle control. These immunizations were performed under light anesthesia. For experiments involving dead spores, spores were autoclaved before protein adsorption. For some experiments, the adjuvant poly(I:C) (Sigma-Aldrich) was used intranasally at a dose of 20 µg.

Mice were infected with approximately 200 *M. tuberculosis* bacilli per animal delivered *via* low-dose aerosol, using a Biaera aerosol generator (Biaera Technologies). Infectious dose was routinely verified by standard plating techniques.

### Bacteria and Colony Forming Unit Quantification

Bacillus Calmette-Guérin Pasteur and Mtb (strain H37Rv) were used for the *in vivo* experiments; these were kind gifts of Professor Juraj Ivanyi (King’s College, London). Both strains were grown to log phase at 37°C in 7H10 broth (Becton Dickinson) supplemented with ADC (Becton Dickinson), 0.05% Tween-80 and Selectab (Mast Diagnostics). Bacteria were then enumerated by the standard CFU method on 7H11 agar plates [supplemented with OADC (Becton Dickinson), glycerol and Selectab (Mast Diagnostics)] and cryopreserved in liquid nitrogen until use.

Bacterial burden from mouse organs was assessed by CFU enumeration. Lung and spleen homogenates were prepared in a stomacher containing 0.1% Triton X-100. Homogenates were plated in technical duplicates (lungs) or singlets (spleens) on 7H11 agar supplemented with OADC, glycerol and Selectatab. CFUs were counted after a 3–4-week incubation at 37°C.

### Vaccines and Immunization

Amino acid sequences of the Mtb proteins ACR, Ag85B (pos. 23–25), and HBHA (pos. 160–199) were connected via linker peptides (GGGSGGGS), and six histidine residues were added to the C-terminus resulting in FP1. The amino acid sequence of FP1 was retranslated to DNA considering the codon usage of *Escherichia coli* DH5α, the host strain for protein production. In order to enable site-directed cloning, restriction sites for *Nco*I and *Hind*III were added to the 5′ and 3′ end, respectively. The synthetic gene was provided by GenScript (USA) inserted in pUC57. The gene of FP1 was excised from this plasmid using the abovementioned restriction endonucleases and ligated to expression vector pLEXWO481, an IPTG-inducible derivative of pMV261 (24), digested with the same enzymes as before. For production of FP1, the gene was expressed under control of lac-promoter while growing the host strain in APS medium at 30°C. Recombinant protein was isolated from inclusion bodies after denaturation in 8 M urea using metal chelate chromatography (Ni-NTA Superflow, Qiagen). Highly enriched FP1 was refolded by gel-filtration using sephadex G-200 material (GE Healthcare). Purity was assessed by fully automated SDS-PAGE with fluorimetric detection and densitometric purity (>97% purity). Western blots specific for component antigens were used to confirm the identity of the protein band. Endotoxin content was measured by LAL assay and determined to be <7 IU/mg. For formulation, FP1 was incubated with spores for 1 h at room temperature prior to the addition of polyI:C (if used). Vaccines were delivered immediately after formulation.

### Antibody and Antigen Quantification

Antibody levels (IgA and IgG) in BAL and serum were quantified by ELISA. Antigens [Ag855, ACR; Lionex GmbH (Braunschweig, Germany)] were coated onto a plate at 2 µg/mL overnight, followed by blocking for 2 h with PBS containing 1% bovine serum albumin (BSA). BAL and serum were diluted 1:250 and 1:1,000, respectively, in PBS with 1% BSA and incubated on the plate in triplicate for 1 h at 37°C. Levels of IgA or IgG were detected using peroxidase-conjugated anti-mouse IgA or anti-mouse IgG (Sigma) and OPD substrate (Sigma). Plates were read on a Tecan200 plate-reader at 450 nm absorbance.

Assessment of protein loading onto spores and stability of final product was done by ELISA (quantifying unbound protein after adsorption) and by measuring charge and size using a ZetaSizer NanoZS (Malvern) according to manufacturer’s instructions and proprietary software. Significance was tested with a paired *t*-test. For ELISA measurements, FP1 was coated onto plates at varying concentrations as described for the antibody measurements, followed by detection by a peroxidase-conjugated anti-His antibody (Sigma) in conjunction with OPD substrate. Plates were read as described above.

### General Flow Cytometry

For most experiments, cells were first stained with Fixable Viability Dye eFluor^®^ 780 (1:1,000 dilution; eBioscience) in the presence of Fc receptor blockade (TruStain, 1:500 dilution; Biolegend). For surface staining, cells were then stained in flow cytometry buffer (PBS containing 0.5% BSA and 0.1% sodium azide—all from Sigma-Aldrich) for 30–45 min at 4°C. For some experiments, cells were subsequently fixed in the appropriate fixative for 30 min at 4°C, and then stained in a permeabilization buffer for 45 min, followed by acquisition on a BD FACSCanto II, unless otherwise specified. For compensation matrices, UltraComp beads were used according to the manufacturer’s instructions (eBioscience). Staining boundaries were determined by a combination of antibody titration, biological controls and fluorescence-minus-one samples.

### Antigen-Presenting Cell (APC) Activation

Dendritic cells (DCs) were obtained according to a well-established protocol (25). Briefly, mouse femurs were aseptically flushed with complete RPMI (RPMI-1640 containing 100 U/mL penicillin/streptomycin, 2 mM l-glutamine, 10% fetal calf serum, and 50 µM 2-mercaptoethanol—all from Sigma-Aldrich) and the bone marrow cells were cultured in complete RPMI with 50 ng/mL GM-CSF (Peprotech) for 2 days, followed by complete removal of the liquid media containing non-adherent granulocytes, and replacement with fresh GM-CSF-supplemented media. Cells were then cultured for a further 3–4 days, and non-adherent and loosely adherent cells were gently detached. DCs were phenotyped by flow cytometry and were found to be>85% CD11c^+^ and expressing high levels of MHC Class II. DCs were cryopreserved in 10% DMSO until use. For experiments involving macrophages, the J774 cell line was used. Macrophages were cultured in complete DMEM (from Sigma, see RPMI), and sub-cultured every 3 days at ~80% confluency. Cells were>99% CD11b^+^ as assessed by flow cytometry.

To measure activation, APCs were stimulated for 48 h with *B. subtilis* spores at an MOI of 1, 10, or 100, or *E. coli* LPS (100 ng/mL; Sigma-Aldrich), and stained with a panel of antibodies: CCR7-PerCP/Cy5.5, CD80-APC, CD86-PE/Cy7, MHC Class I-FITC, MHC Class II-Brilliant Violet 510, PD-L1-Brilliant Violet 421, and PD-L2-PE—all from Biolegend. Supernatants were tested for IL-1β, IL-6, and TNF-α. IL-12p40 was detected by intracellular cytokine staining after 20 h stimulation of macrophages in the presence of 10 µg/mL brefeldin A (Sigma-Aldrich) using IL-12p40-PE (Biolegend) in flow cytometry buffer containing 0.5% saponin (Sigma-Aldrich). To detect transcription factor phosphorylation, macrophages were stimulated for 4 h and then fixed in 90% methanol as previously described (26), followed by staining with antibodies against phosphorylated forms of c-Jun (AP-1), NF-κB (p60), and IRF-3 (Cell Signaling).

### T-Cell Proliferation

Splenocytes were obtained from mouse spleens that had been mechanically homogenized and treated with ACK lysis buffer (Sigma-Aldrich) to remove erythrocyte contamination. Splenocytes were cultured at 1 × 10^6^/well in a 96-well plate. Cells were stimulated with 5 µg/mL recall antigen or 1 µg/mL α-CD3 (Biolegend) for 5–6 days, followed by surface staining with CD4-PerCP/Cy5.5, CD8-Brilliant Violet 510, CD44-FITC, CD62L-PE, and CD90.2-Brilliant Violet 421—all from Biolegend. Cells were then fixed and permeabilized using the eBioscience Foxp3/Transcription Factor Staining Buffer Set and stained with Ki67-APC.

### Cytokine Quantification

Cytokines were measured by ELISA or Multiplex immunoassay. IL-1β, IL-6, and TNF-α in culture supernatant were measured by ELISA using eBioscience Ready-Set-Go kits according to the manufacturer’s instructions. For IL-4, IL-10, IL-17A, and IFN-γ, a 4-plex multiplex immunoassay (Biolegend) was used according to manufacturer’s instructions. Data was acquired on a BD FACSCanto, and analysis performed using Legendplex software (Biolegend).

### Lung Cell Isolation and Analysis

For these experiments, mice were immunized with PBS or BCG (subcutaneously) or mucosally with the indicated vaccine component. Lungs were perfused of blood by flushing PBS through the right ventricle. Tissue was then dissected into 1 mm pieces using a scalpel, followed by digestion in 1 mg/mL collagenase and 0.5 mg/mL DNase I (Roche). Cells were then passed through a 70 µm strainer (Becton Dickinson), contaminating erythrocytes were lysed, and mononuclear cells were stained for CD3-APC, CD4-PerCP/Cy5.5, CD8-Brilliant Violet 510, CD44-FITC, CD62L-PE, CD69-PE/Cy7, and CD103-Brilliant Violet 421—all from Biolegend.

### Statistical Analysis

Statistical tests are described in the relevant figure legends. All analysis was performed using FlowJo v10, Microsoft Excel 2010 and GraphPad Prism 7.

## Results

### Spores Can Effectively Bind to Mtb Fusion Proteins

We tested whether a biological carrier system, such as spores, could bind and facilitate carriage of the FP-1 fusion-protein. Spores were incubated with FP-1 prior to centrifugation to quantity the amount of free FP1 present in the supernatant after adsorption. Of 100 µg FP1, less than 2 µg free FP1 was detected in supernatants after adsorption indicating a high binding efficiency of over 98% (*n* = 3). To characterize the formulated Spore-FP1 vaccine, we assessed the size and charge of Spores either pre- or postadsorption using a ZetaSizer NanoZS. Size of the spores increased following FP-1 adsorption from 1,337 ± 13.8 to 1,389 ± 13.53 nm for naked and loaded spores, respectively (*n* = 3). Spores became marginally more negative when FP-1 was loaded, with charge decreasing from −47.13 ± 0.95 to −49.1 ± 0.44 mv (*n* = 3). While neither of the changes were significant, both trended toward significance (*p* = 0.064 and 0.068 for charge and size, respectively) and, in conjunction with the ELISA data, are suggestive of protein loading onto the spore surface. Importantly, FP1 loaded spores were moderately negatively charged and had a low polydispersity index (PDI = 0.237), indicating strong colloidal stability.

### Spore-FP1 Can Enhance Bacterial Control Afforded by BCG

**Figure 1:**
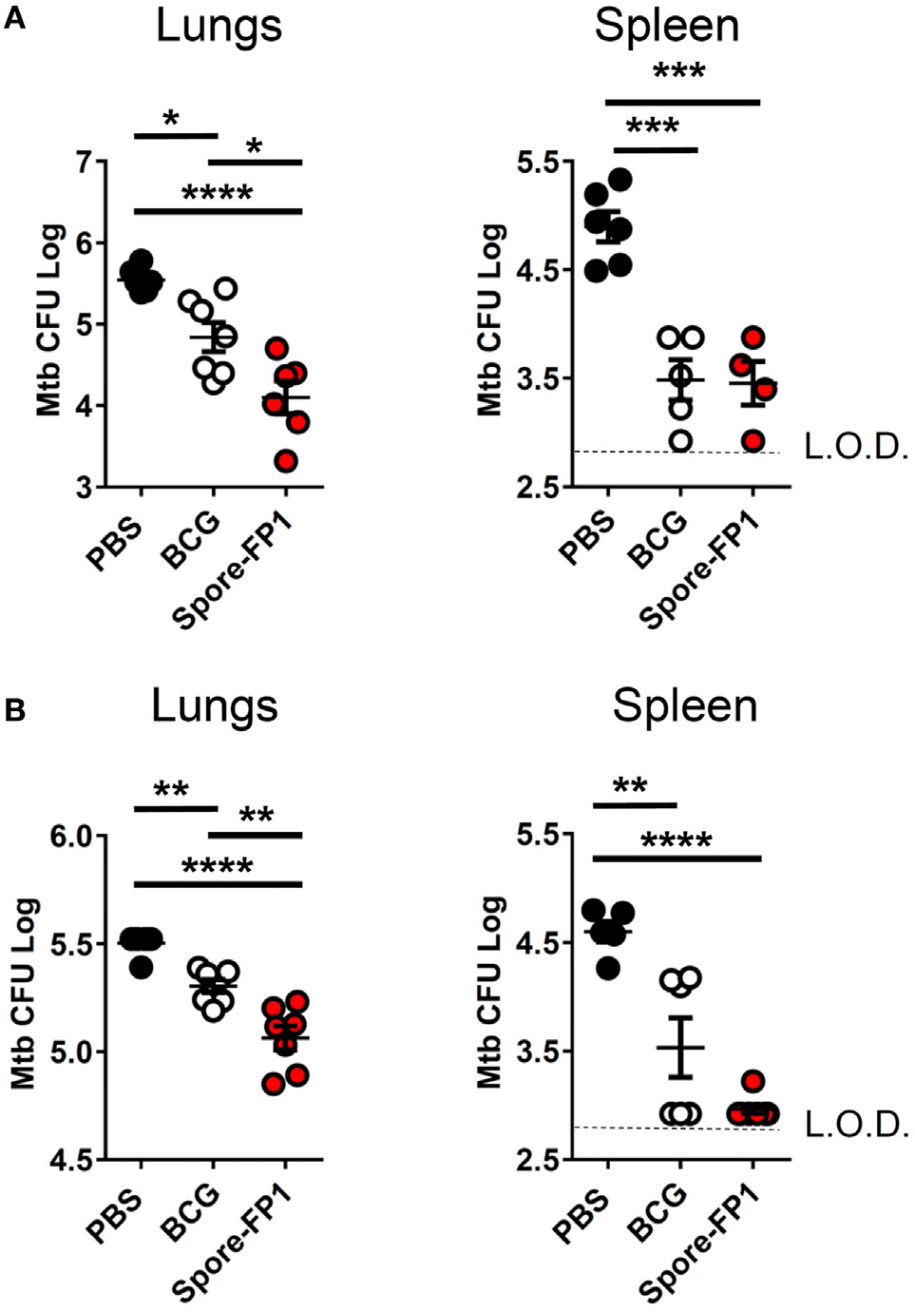
Spore-FP1 protects against aerosol *Mycobacterium tuberculosis* (Mtb) challenge. Mice received a Bacillus Calmette-Guérin subcutaneous prime (except the PBS control group) followed by two intranasal boosts with Spore-FP1. After 3 weeks, bacterial burdens in the lungs and spleens were quantified by CFU counting on 7H11 plates across three dilution ranges. **(A)** Mice were immunized with Spore-FP1 alone. **(B)** Mice were immunized with Spore-FP1 in combination with the adjuvant poly(I:C). Results are expressed as mean ± SEM. Data are derived from *n* = 4–7 individual mice. Significance was tested against the by one-way ANOVA with Tukey’s posttest, *p < 0.05, **p < 0.01, ***p < 0.001, and ****p < 0.0001.

Mucosal vaccination against respiratory diseases offers distinct advantages over parenteral delivery routes, such as enhanced control of the pathogen, presumably due to localized immune effector cells (27). We therefore tested the ability of mucosally-delivered Spore-FP1 to enhance control of Mtb, compared to BCG immunization alone, in a “prime-boost” strategy. We hypothesized that a booster immunization with Spore-FP1 would lead to better protection than immunization with BCG alone. Mice were first primed with BCG or vehicle control, followed by two intranasal boosts with Spore-FP1. Mice were then challenged with ~100 CFU aerosolized Mtb, and bacterial burdens were quantified in the lungs and spleen. As shown in Figure 1A, mice immunized with BCG alone were better able to control Mtb compared to mock-immunized mice, as evidenced by reduced CFUs in the lungs (PBS CFUs: 5.54 ± 0.06; BCG CFUs: 4.84 ± 0.18, *p* < 0.05). When mice received the Spore-FP1 booster immunization, however, there was a significant improvement compared to BCG alone, with a near −1 log reduction in bacterial burden (Spore-FP1 CFUs: 4.10 ± 0.20, *p* < 0.0001 vs. PBS, *p* < 0.05 vs. BCG). In the spleens, BCG and Spore-FP1 offered comparable levels of protection, with both groups exhibiting significant protection compared to mock immunization (*p* < 0.0001). Next, Spore-FP1 was tested with codelivery of the adjuvant poly(I:C), a known inducer of the Th1 subset when used in the respiratory tract (28). As can be seen in the lungs (Figure 1B), Spore-FP1 was again able to induce significantly better protection than BCG (*p* < 0.01), with a trend—though not a statistically significant difference—for increased protection in the spleen (*p* = 0.06, BCG vs. Spore-FP1). These data collectively demonstrate that Spore-FP1 immunization could improve protection offered by BCG alone in multiple contexts, and therefore we sought to uncover any immunological phenomena that could be associated with efficacy.

### Spore-FP1 Enhances Antigen-Specific Antibody Production

**Figure 2:**
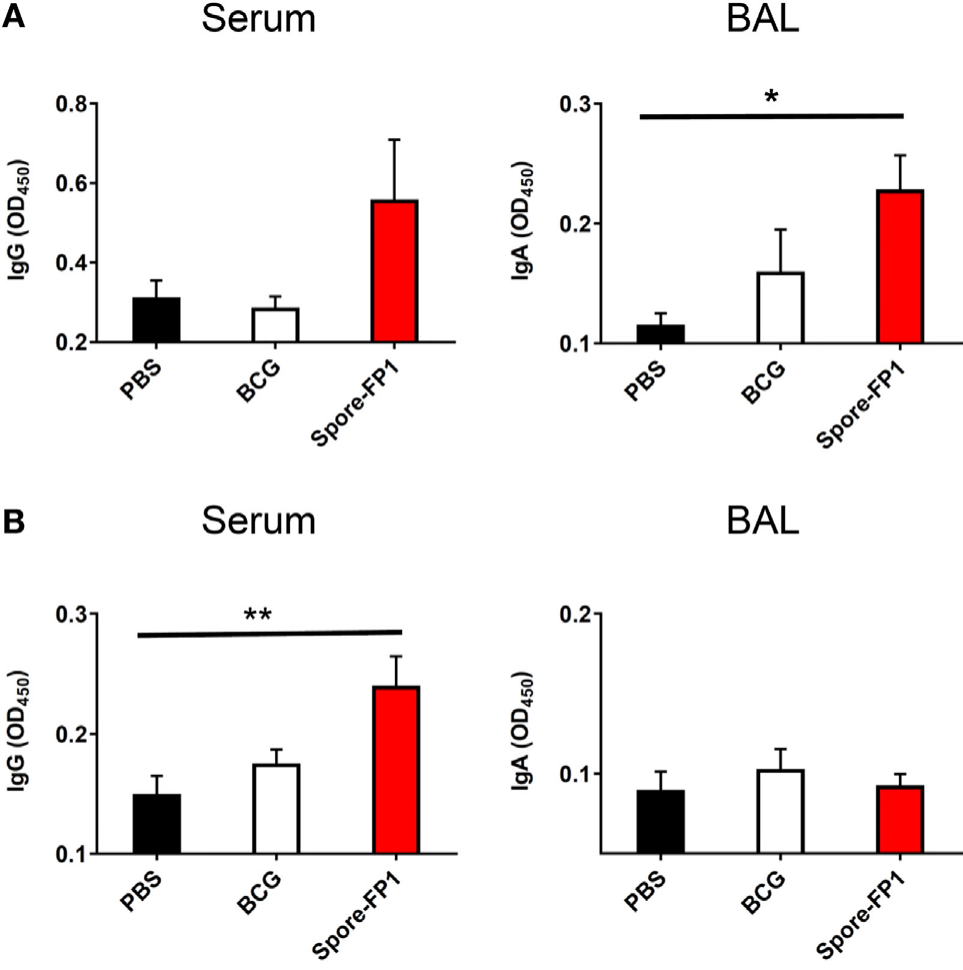
Enhanced humoral immunity caused by Spore-FP1. Immunized mice were tested for the presence of antigen-specific IgG in the serum (1:1,000 dilution) and IgA in the BAL (1 mL PBS flush; 1:10 dilution) by ELISA, with optical density read at 450 nm in duplicate. **(A)** Levels of IgG and IgA specific to Ag85B. **(B)** Levels of IgG and IgA specific to ACR. Results are expressed as mean ± SEM. Data shown are derived from *n* = 3 individual mice and are representative of two independent experiments. Significance was tested against the unstimulated control by one-way ANOVA with Tukey’s posttest, *p < 0.05 and **p < 0.01.

Evidence suggests that antibodies may play a role in protecting against TB, either directly (Fab-mediated) or indirectly (Fc-mediated): monoclonal IgA therapy can reduce pulmonary Mtb burden (29), and adoptive transfer of antibodies from hosts with latent TB can improve macrophage functionality (30). We therefore probed whether Spore-FP was generating antibodies against Ag85B and ACR. Spore-FP1 immunization significantly enhanced titers of Ag85B-specific IgA in the BAL compared to PBS (*p* < 0.05), whereas there was no difference with BCG (Figure 2A). There was also a trend for increased levels of Ag85B-specific IgG induced by Spore-FP1. With regards to ACR-specific antibodies (Figure 2B), Spore-FP1 was able to significantly enhance levels of α-ACR IgG in the serum, compared to PBS (*p* < 0.01). However, there were no changes in the α-ACR IgA within the BAL.

### Spore-FP1 Generates Abundant Tcm and Tem Cells with High Proliferative Capacity

**Figure 3:**
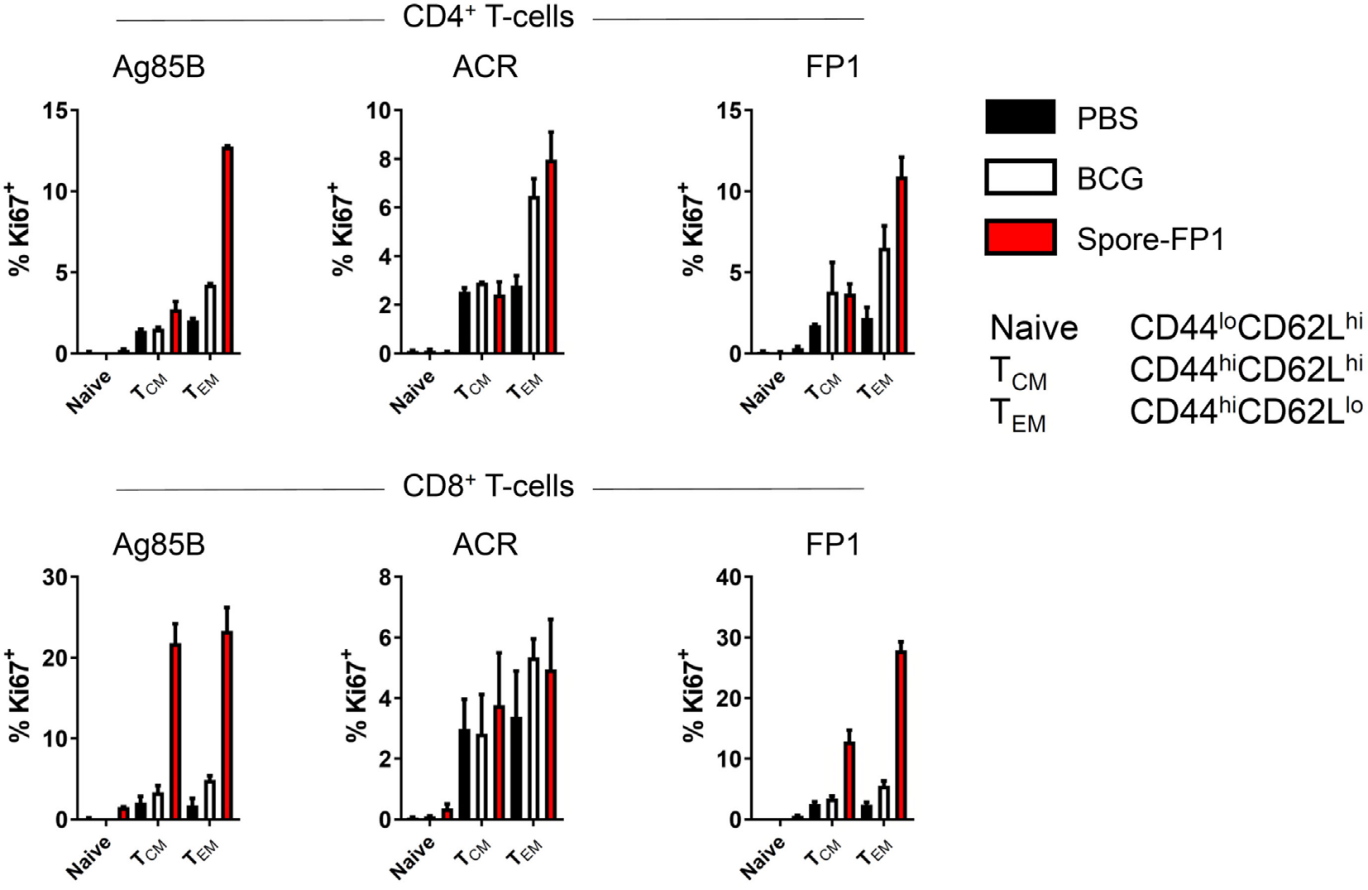
Enhanced T-cell proliferation due to Spore-FP1. Splenocytes were incubated in technical duplicates with 5 µg/mL recall antigen for 5–6 days and proliferation was measured by Ki67 staining. A gating strategy of live cells→single cells→CD3^+^→CD4^+^/CD8^+^ was used, followed by gating for Ki67^+^ cells and determination of memory cell phenotype by expression of CD44 and CD62L. Results are expressed as mean ± SEM. Data are derived from *n* = 3 pooled spleens per group.

The observation that Spore-FP1 immunization led to higher Mtb-specific IgG and IgA titers suggested that T-cell immunity was also being modulated. We hypothesized that Spore-FP1 was inducing stronger T-cell immunity than BCG alone, leading to enhanced antibody levels. To test this, splenocytes from immunized mice were assessed for the expression of the cell cycle and proliferation marker Ki67 after exposure to the recall antigens Ag85B, ACR and FP1. The Ki67^+^ cells were then divided into naive (CD44^lo^CD62L^hi^), T central memory (Tcm; CD44^hi^CD62L^hi^) or T effector memory (Tem; CD44^hi^CD62L^lo^) phenotypes. As shown in Figure 3, as expected, there was minimal proliferation in the PBS group in response to all antigens, with a background level of ~3% Ki67^+^ in memory cell subsets. There were modestly more proliferating cells in the BCG group, which is consistent with other studies showing that BCG induces a very small percentage of antigen-specific splenic T-cells (31, 32). For instance, there were 6.48% Ki67^+^ CD4^+^ Tem cells after ACR stimulation in this group, and a similar level in the CD8^+^ Tem cells. However, in the Spore-FP1 group, there was a sharp overall increase in the percentage of Ki67^+^ cells, with notable spikes (>20%) in proliferating CD8^+^ Tcm and Tem cells in response to Ag85B. Similarly, Spore-FP1 had the highest percentage of CD4^+^ Tem cells responding to Ag85B (>10%). Results for ACR in this group were more modest, but the trend remained consistent. These data support the ability of a mucosal vaccine to induce substantial T-cell responses at primary lymphoid sites.

### Spore-FP1 Immunization Results in a Mixed T-Cell Cytokine Profile

**Figure 4:**
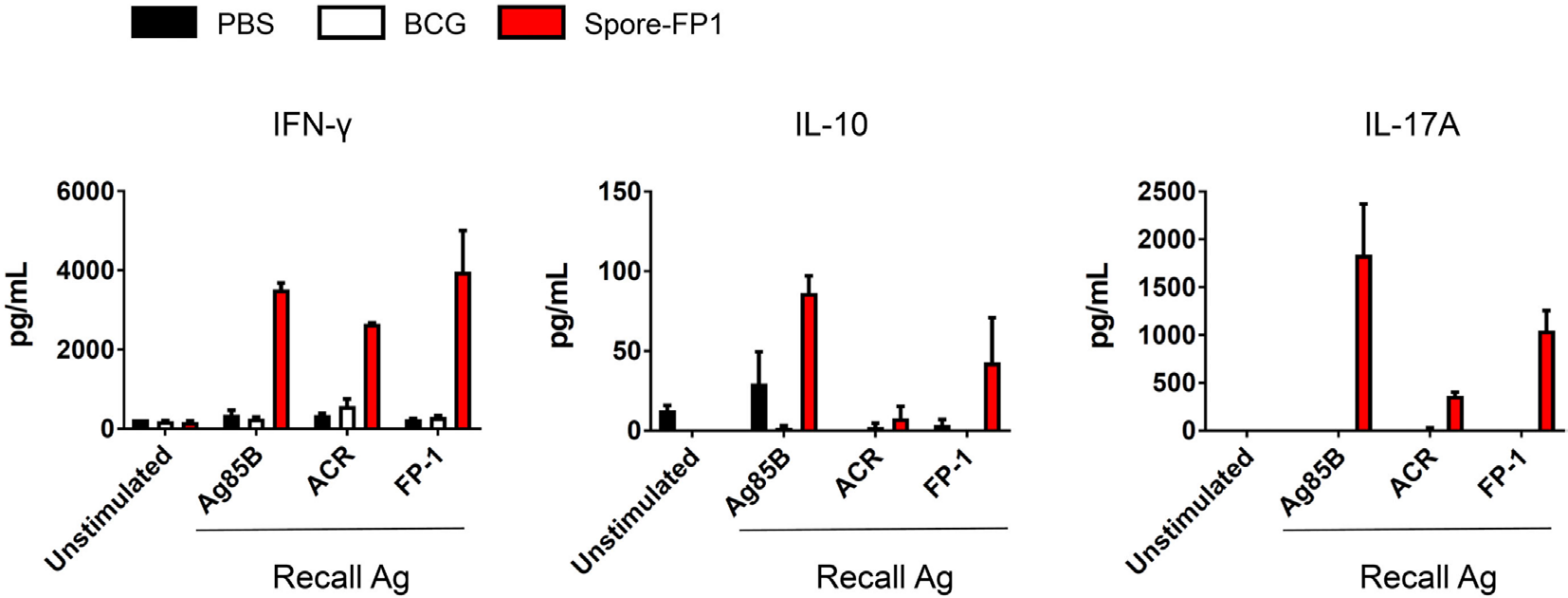
Cytokine profiles during splenocyte antigen recall. Splenocytes from immunized mice were stimulated in technical duplicates with 5 µg/mL recall antigen for 5–6 days and T-cell cytokines were measured by multiplex flow cytometry. Results are expressed as mean ± SEM. Data are derived from *n* = 3 pooled spleens per group.

The increase in T-cell proliferation in response to mucosal immunization with Spore-FP1 led us to question which subsets of T helper cells and cytotoxic T-cells were responding to antigen. Therefore, splenocytes from immunized animals were cultured with recall antigens (Ag85B, ACR and FP1) and assessed for the production of IFN-γ, IL-4, IL-10, and IL-17A, which are secreted from Th1/Tc1, Th2/Tc2, Treg, and Th17/Tc17 subsets, respectively. We found (Figure 4) that there was muted cytokine production across all analytes in the BCG group when cells were stimulated with recall antigens, with the exception of minor IFN-γ secretion (570.36 pg/mL) after ACR pulsing. In the Spore-FP1 group, however, there was profound cytokine release in response to all three antigens. After Ag85B pulsing, Spore-FP1 splenocytes produced copious amounts of IFN-γ (3519.6 pg/mL), IL-10 (86.26 pg/mL), and IL-17A (1837.5 pg/mL), suggesting that Spore-FP1 immunization generated mixed T-cell subsets that were specific for Mtb antigens. Similar results were observed for FP1 antigen recall, and there were modest levels of cytokines for ACR. No IL-4 was detected in any of the groups.

### Evidence of Tissue-Resident Memory T-Cells after Mucosal Immunization

**Figure 5:**
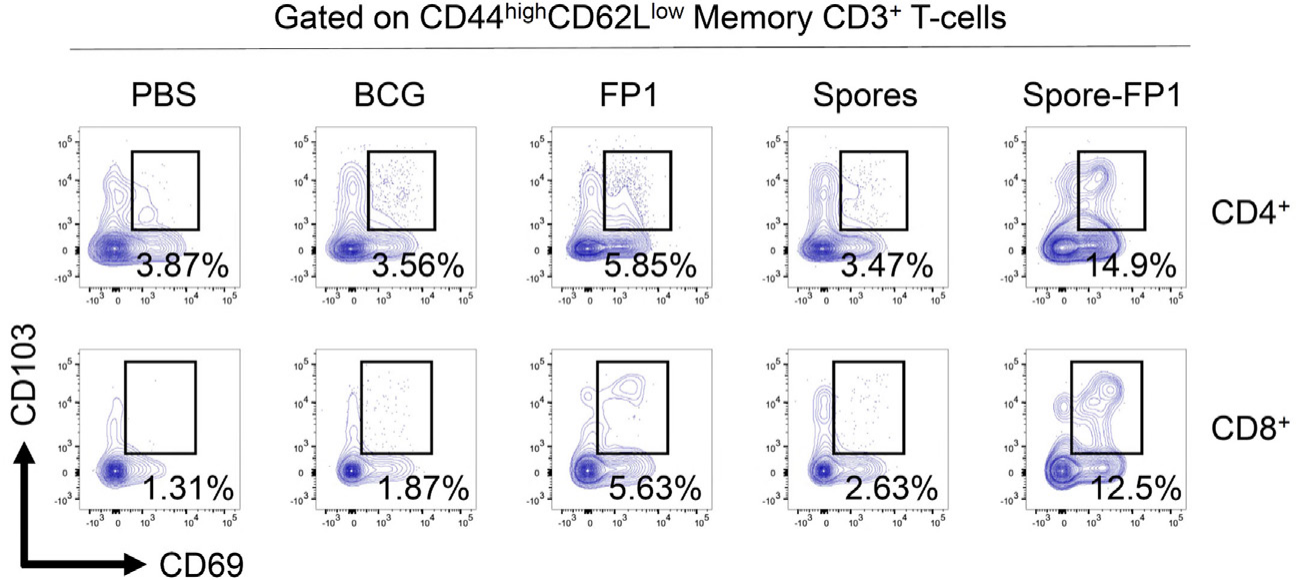
Spore-FP1 induces enrichment of tissue resident memory cells. Mice were first immunized with Bacillus Calmette-Guérin for 6 weeks (except the PBS group) and then received two intranasal doses of either spores alone, fusion protein 1 (FP1) alone, or Spore-FP1. Lung parenchymal cells were assessed by flow cytometry for T-cell markers. A gating strategy of live cells→single cells→CD3^+^→CD4^+^/CD8^+^→CD44^hi^CD62L^lo^ was used to measure the frequency of double-positive CD69/CD103 Trm. Data are derived from *n* = 3 pooled mice per group showing a representative plot.

Since Spore-FP1 was causing the development of antigen-specific T-cells in central lymphoid organs, we next interrogated the lungs for the presence of tissue-resident memory T-cells. Lungs were perfused and harvested from immunized animals, and then CD44^hi^ (i.e., memory) T-cells were assessed for the expression of tissue retention markers CD69 and CD103. As shown in Figure 5, PBS and BCG immunization induced minimal levels of these cells (<4% in both CD4^+^ and CD8^+^ T-cells), with only a minor increase induced by FP1 alone. Notably, the mucosal delivery of *B. subtilis* spores alone did not lead to the generation of Trm, while the full vaccine construct, Spore-FP1, was able to induce 14.9% CD4^+^ and 12.5% CD8^+^ Trm, respectively.

### Bacillus Spores Activate Macrophages and DCs

**Figure 6:**
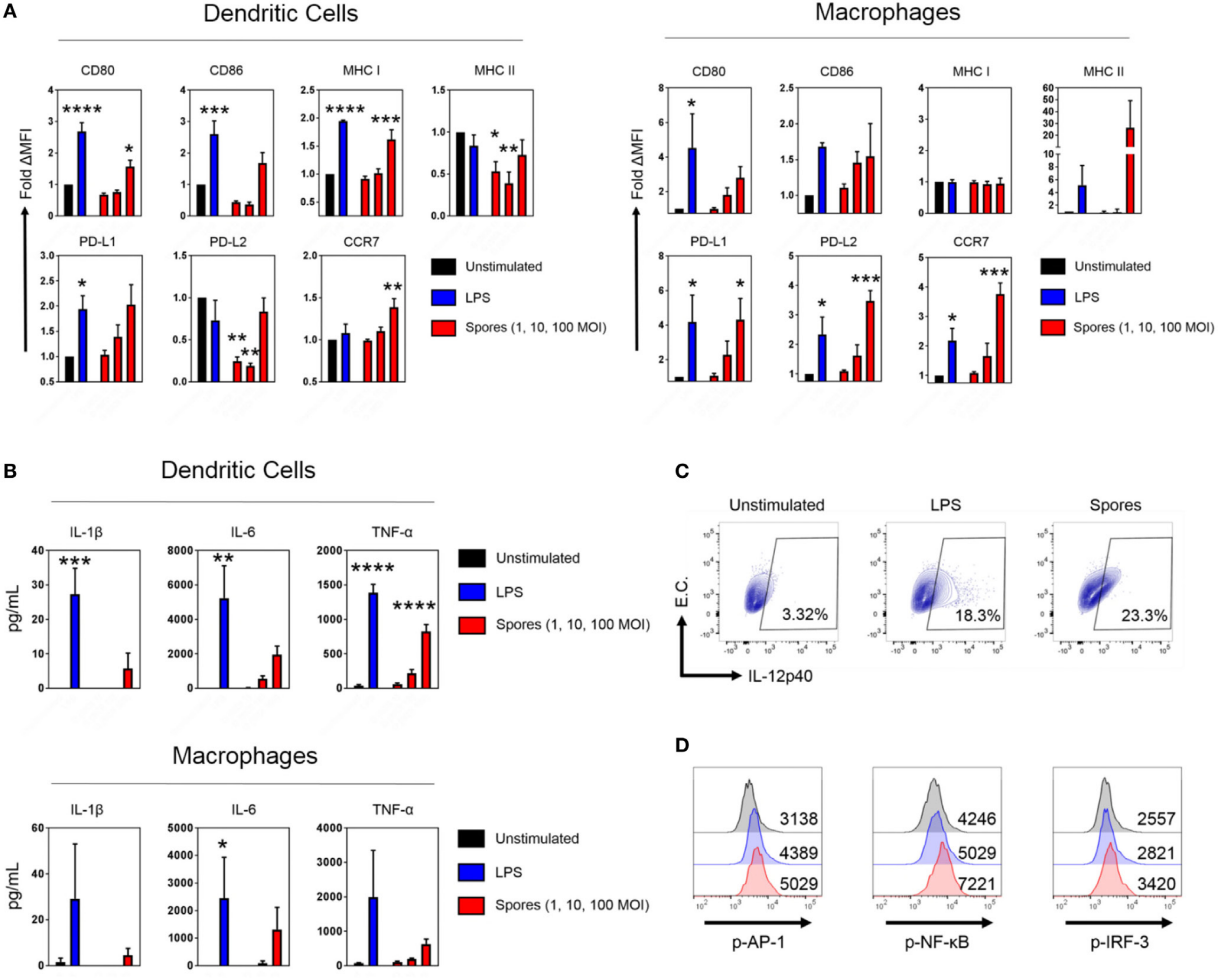
*Bacillus subtilis* spores activate antigen-presenting cells. **(A)** Dendritic cells (DCs) (left) and macrophages (right) were stimulated in duplicate for 48 h with LPS (100 ng/mL) or *B. subtilis* spores (1, 10, and 100 MOI) and surface molecule expression was measured by flow cytometry on gated viable cells. MFI was normalized to the unstimulated control. **(B)** Cytokines from the supernatants were tested for proinflammatory cytokine production by ELISA. **(C)** Macrophages were stimulated for 20 h with LPS (100 ng/mL) or *B. subtilis* spores (100 MOI) in the presence of brefeldin A (10 µg/mL), followed by intracellular detection of IL-12p40. EC, empty channel. A representative experiment is shown. **(D)** Transcription factor phosphorylation levels were determined by PhosphoFlow. Macrophages were stimulated with 100 ng/mL LPS (blue histograms) or 100 MOI spores (red histograms) for 4 h and then fixed and stained. Some cells were left untreated (black histograms). Representative MFI values are plotted on the relevant histogram. Data are from three **(A–C)** or one **(D)** independent experiments. Results are expressed as mean ± SEM. Significance was tested against the unstimulated control by one-way ANOVA with Fisher’s posttest, *p < 0.05, **p < 0.01, ***p < 0.001, and ****p < 0.0001.

Antigen-presenting cells are essential for the generation of T-cell immunity after immunization (33, 34). Empirical and systems biology approaches have revealed a correlation between APC activation by some antibody-inducing vaccines and protective immunity (35–37). Consequently, we next tested whether *B. subtilis* spores could activate DCs and macrophages (Figure 6). DCs and macrophages were pulsed with *B. subtilis* spores for 2 days at a range of MOIs and assessed for the upregulation of maturation markers. In DCs, spores significantly upregulated CD80, MHC Class I and CCR7 (CD80: *p* < 0.05, MHC Class I: *p* < 0.001, CCR7: *p* < 0.01), with strong trends for upregulation of CD86 and PD-L1 (Figure 6A). Interestingly, spores induced the downregulation of MHC Class II and PD-L2. This may reflect the time-point at which the markers were measured, since Class II is known to be upregulated initially, followed by late-phase downregulation in activated DCs (38). In macrophages, spores were able to induce the upregulation of PD-L1 (*p* < 0.05), PD-L2 (*p* < 0.001), and CCR7 (*p* < 0.001), with similar trends for CD80, CD86, and MHC Class II. With regards to the production of proinflammatory cytokines, there was evidence of secretion of IL-6 and TNF-α at an MOI of 100 in DCs and macrophages (Figure 6B), albeit at lower levels compared to the positive control LPS. There was modest IL-1β production at the highest dose of spores. Next, we used intracellular cytokine staining to detect IL-12p40 production after spore exposure (Figure 6C). Spores were found to induce comparable levels of IL-12p40^+^ APCs (23.3%) compared to LPS (18.3%). As expected, there was minimal production of IL-12p40 in unstimulated cells. Finally, we investigated three transcription factors downstream of Toll-like receptors (TLRs) to understand the cause of the phenotype. Intracellular flow cytometry at 4 h poststimulation (Figure 6D) revealed that *B. subtilis* spores were activating AP-1 (c-JUN), NF-κB and IRF-3 to a greater extent than LPS, suggesting engagement of both MyD88 and TRIF adaptors, in tandem with mitogen-activated protein kinase activity.

## Discussion

Tuberculosis is a disease defined by an immunological complexity that has hindered efforts toward vaccine development and allowed Mtb to persist perniciously across the globe. The cause of this complexity is a raft of exquisite immune evasion mechanisms that has evolved over thousands of years to manipulate host immunity. Therefore, at present, animal infection models are the most useful tool for predicting TB vaccine success (10).

We have previously reported that spores coated with Mtb antigens can provide good protection against Mtb in an intranasal infection model (21). A major limitation of this model, however, is that intranasal infection is not physiologically representative of natural Mtb infection. Furthermore, this study did not address whether such vaccine constructs could enhance pre-existing BCG-mediated immunity in the mouse model in a prime-boost immunization schedule. In the present study, we used a technologically superior low-dose aerosol Mtb challenge model to test the efficacy of a novel TB vaccine, Spore-FP1, on BCG-immunized mice, with the hypothesis that Spore-FP1 could boost the protection afforded by BCG. Spore-FP1 was designed to include antigens expressed during the early (Ag85B) and late (ACR) phases of Mtb infection, which could help to account for some of the observed dynamic changes in antigen expression during disease (39). Moreover, unlike previous studies, Spore-FP1 also contained a portion of the epithelium-binding domain of HBHA, which would encourage attachment to lung epithelium and dissemination of the vaccine (40).

Our most important finding was that intranasal immunization with Spore-FP1 in a BCG-primed mouse (i.e., the “prime-boost” strategy) could significantly enhance protection above that given by BCG alone. This effect was independent of any coadjuvants or whether the spores were viable or not. Bacterial burdens in the lungs were significantly reduced by the Spore-FP1 booster immunization compared to BCG alone. Interestingly, there was no statistically significant reduction in extra-pulmonary Mtb. This may reflect a limitation of detection in our assays, or at least an area of potential development for future utilization of spore vehicle systems. Distinct T-cell-derived cytokine patterns are known to exert dichotomous effects on bacterial burdens in the lungs and spleen (41), which may be the case here. Furthermore there was also a slight reduction in protective effect when Spore-FP1 was used with adjuvant. This may reflect ordinary biological variation between experiments, or perhaps non-optimal adjuvant choice, and will be the subject of future investigations.

To investigate immunological events associated with protection, we first evaluated antigen-dependent antibody production in serum and BAL. The role of antibodies in TB is contentious, although there have been recent reappraisals of the field generally in favor of their protective role (42). Mice immunized with Spore-FP1 were found to produce more Ag85B-specific IgG in the serum and IgA in the BAL than the BCG group; a similar trend was observed for ACR. These data strongly suggest that a Spore-FP1 boost immunization was better at inducing humoral immunity than a single BCG immunization.

T-cells are essential for protection against Mtb: Th1 cells prime macrophages for activation via IFN-γ (43), and Th17 cells can upregulate the production of antimicrobial peptides and lymphocyte chemoattractants (44, 45). Deficiency in either of these cytokines is extremely detrimental to the host during disease. It has been shown that during natural infection, Mtb can subvert the host immune system in order to restrict antigen presentation (46, 47). Hence a vaccine that enhances antigen presentation, and thus leads to a higher frequency of antigen-specific T-cells, is highly desirable. In our experiments, we observed a higher frequency of proliferating splenic T-cells in response to recall antigens in the Spore-FP1 group compared to mice that had only received BCG immunization. BCG is also able to restrict antigen presentation *in vivo* to a certain extent (47–49), and consistent with this fact, we observed minimal proliferative responses to Ag85B/ACR in BCG-immunized animals. Such small-magnitude responses in BCG-immunized mice are highly typical and described elsewhere in the literature, wherein cells specific for Ag85 typically represent ~0.1% of the total splenic polyclonal T-cell pool in cytokine capture assays (31, 32). In contrast to these constrained responses, Spore-FP1 was able to induce a dramatically larger percentage of proliferating T-cells, indicating either a higher frequency of memory cells, or at the very least cells with a higher proliferative capacity. Many of the proliferating CD8^+^ Ki67^+^ cells were of the Tcm phenotype, which act as a “reservoir” of cells in primary and secondary lymphoid organs with high potential for differentiation into effector cells in distal sites. For chronic diseases such as TB that include T-cell exhaustion as a definitive mechanism of immune evasion (i.e., terminal differentiation), the generation of proliferative Tcm by a prophylactic vaccine offers a distinct advantage.

In line with proliferative responses, Spore-FP1 was also a potent inducer of IFN-γ, IL-10, and IL-17A release after splenocyte exposure to recall antigens. Thus, the antigen-specific cells were fully functional by producing effector cytokines during proliferation. It could be surmised that Spore-FP1 therefore induced a mixed Th1-Th17-Treg response. The absence of IL-4 release is interesting, and suggests that Spore-FP1 induced a T-cell skewing away from the Th2 to a Th1/Th17 phenotype. IL-4 is largely believed to be detrimental during Mtb infection, since it antagonizes the biological effects of IFN-γ to promote alternatively activated macrophages (50). The role of IL-10 in TB is more contentious. While IL-10 can hamper antimycobacterial immunity during BCG immunization (51), recent evidence from *Rhesus macaque* infection models has suggested that CD4^+^ T-cells coexpressing a balance of pro- and anti-inflammatory cytokines are significantly associated with granuloma sterilization, possibly due to a reduction in “collateral damage” to the lung tissue (52). Furthermore, IL-10 is important for shielding CD8^+^ memory T-cells from apoptosis in inflammatory contexts (53), and IL-10 deficient mice are highly susceptible to reinfection by intracellular pathogens (54). We believe that the T-cell profile induced by Spore-FP1 is therefore beneficial in the context of immunization.

It is worth noting that for both humoral and cellular immunogenicity, there was generally a greater response to Ag85B than to ACR. This is perhaps due to the fact that Ag85B is a strong immunodominant antigen (55) that has formed the basis of many new TB vaccines. Notably, however, ACR was still able to elicit potent IFN-γ production in splenocytes from Spore-FP1-immunized mice.

Alongside conventional T-cell activation signatures, we also observed a striking accumulation of gross CD69^+^CD103^+^ Trm in lung tissue after immunization with Spore-FP1. These cells are likely to be directed toward epitopes found within FP1, since the vehicle control (spores alone) failed to induce any appreciable quantities of these cells. As to why no Trm were directed against *B. subtilis* spores themselves, it may be that *B. subtilis*, as a mammalian commensal (56) (in the absence of a “foreign” antigen such as those included in FP1), can suppress the mobilization of effector T-cells that would lead to its own clearance. In support of this hypothesis, *B. subtilis* secretory products can induce a Foxp3-dependent tolerogenic environment in the gut (57), and consistent with this fact, we observed modest IL-10 responses from splenocytes exposed to recall antigen (although much lower than IFN-γ and IL-17A). A recent study has elegantly demonstrated that mucosal immunization with BCG—as opposed to parenteral immunization—leads to the accumulation of Trm in the pulmonary tissue (27). These cells are sufficient for protection, since adoptive transfer of Trm into BCG-naive mice protects against Mtb challenge. We speculate that the enrichment of this cell type in the lungs, induced by Spore-FP1 in our experiments, is playing a major role in the protection afforded by our novel vaccine.

Turning our attention to the innate immune system, we detected potent activation signatures in macrophages and DCs pulsed with *B. subtilis* spores. While it is known that *B. subtilis* spores can activate TLR-2→MyD88 downstream pathways, these studies have largely restricted maturation marker analysis to CD40 and MHC Class I and II expression on DCs (19, 58). Here, we showed for the first time that spores can also simultaneously induce CCR7, PD-L1 and PD-L2 upregulation. Since minimal T-cell priming occurs in the lung (59, 60), CCR7 expression will be critical for DCs that have taken up Spore-FP1 to migrate to the lung-draining lymph nodes and present antigen to naive T-cells. The upregulation of PD-L1 and PD-L2, on the other hand, may mitigate the overall inflammatory response, which is an important boon for mucosal delivery. In justification of this notion, PD-L1 blockade during antigen delivery into the lungs leads to exacerbated irritation and inflammation via Treg depletion, which is ameliorated upon immune reconstitution (61). Underscoring all of these phenotypic characteristics was the observation that IRF-3 was phosphorylated alongside NF-κB upon APC stimulation with spores. These data allude to a novel activation pathway besides the TLR-2→MyD88 axis, which is driving APC activation by *B. subtilis* spores, and has hitherto remained unexplored. This proposition warrants further biochemical investigation.

To conclude, we have shown that Spore-FP1 can enhance protection offered by BCG and also activate multiple arms of the innate and adaptive immune systems. These data demonstrate the potential applicability of Spore-FP1 as a TB vaccine, but also offer fresh insights into the mechanisms of *B. subtilis* spores as a vaccine development platform.

## Ethics Statement

The animal work was reviewed and approved by St George’s University of London Ethics Committee for animal experimentation and studies performed under a valid UK Home Office Project Licence.

## Author Contributions

AC, PH, and GD performed most of the immunization and MTB challenge experiments. SH and ACT performed *in vitro* immunogenicity experiments. MS provided recombinant proteins. SC provided spores. MP performed immunological evaluations. RR conceived the study and wrote up the manuscript with AC.

## Conflict of Interest Statement

Author MS was employed by company Lionex. All other authors declare no competing interests.
